# Association between Antibiotic Exposure and Systemic Immune Parameters in Cancer Patients Receiving Checkpoint Inhibitor Therapy

**DOI:** 10.3390/cancers14051327

**Published:** 2022-03-04

**Authors:** Mitchell S. von Itzstein, Amrit S. Gonugunta, Thomas Sheffield, Jade Homsi, Jonathan E. Dowell, Andrew Y. Koh, Prithvi Raj, Farjana Fattah, Yiqing Wang, Vijay S. Basava, Shaheen Khan, Jason Y. Park, Vinita Popat, Jessica M. Saltarski, Yvonne Gloria-McCutchen, David Hsiehchen, Jared Ostmeyer, Yang Xie, Quan-Zhen Li, Edward K. Wakeland, David E. Gerber

**Affiliations:** 1 Department of Internal Medicine, Division of Hematology-Oncology, University of Texas Southwestern Medical Center, Dallas, TX 75390, USA; mitchell.vonitzstein@phhs.org (M.S.v.I.); jade.homsi@utsouthwestern.edu (J.H.); jonathan.dowell@utsouthwestern.edu (J.E.D.); david.hsieh@utsouthwestern.edu (D.H.); 2 Harold C. Simmons Comprehensive Cancer Center, University of Texas Southwestern Medical Center, Dallas, TX 75390, USA; farjana.fattah@utsouthwestern.edu (F.F.); vijay.basava@utsouthwestern.edu (V.S.B.); jessica.saltarski@gmail.com (J.M.S.); yvonne.gloria-mccutchen@utsouthwestern.edu (Y.G.-M.); yang.xie@utsouthwestern.edu (Y.X.); 3 School of Medicine, University of Texas Southwestern Medical Center, Dallas, TX 75390, USA; amrit.gonugunta@utsouthwestern.edu (A.S.G.); vinita.popat@utsouthwestern.edu (V.P.); 4 Department of Population and Data Sciences, University of Texas Southwestern Medical Center, Dallas, TX 75390, USA; thomas.sheffield@utsouthwestern.edu (T.S.); yiqing.wang@utsouthwestern.edu (Y.W.); jared.ostmeyer@utsouthwestern.edu (J.O.); 5 Department of Pediatrics, University of Texas Southwestern Medical Center, Dallas, TX 75390, USA; andrew.koh@utsouthwestern.edu; 6 Department of Immunology, University of Texas Southwestern Medical Center, Dallas, TX 75390, USA; prithvi.raj@utsouthwestern.edu (P.R.); shaheen.khan@utsouthwestern.edu (S.K.); quan.li@utsouthwestern.edu (Q.-Z.L.); edward.wakeland@utsouthwestern.edu (E.K.W.); 7 Department of Pathology, University of Texas Southwestern Medical Center, Dallas, TX 75390, USA; jason.park@utsouthwestern.edu

**Keywords:** antibiotics, antibodies, biomarkers, cancer, cytokines, efficacy, immune checkpoint inhibitors, immunotherapy

## Abstract

**Simple Summary:**

Patients treated with antibiotics have worse outcomes from cancer immunotherapy. While antibiotics are known to affect intestinal flora, it is not clear how they affect overall immune function. The aim of our study was to identify differences in immune parameters according to antibiotic exposure. Among 251 total patients, the 135 (54%) who received antibiotics had lower response rates and shorter survival. We identified significant differences in multiple antibodies according to antibiotic exposure, including antibodies specific for nucleolin, MDA5, c-reactive protein, LC1, heparin sulfate, Matrigel, and CENP.B. In lung cancer patients, antibiotics were associated with differences in IFN-γ, IL-8, and macrophage inflammatory protein cytokines. Administration of antibiotics to patients receiving cancer immunotherapy is associated with changes in circulating antibodies and cytokines, although it is not clear if antibiotics cause these differences. Given the frequency of antibiotic use in cancer populations and potentially detrimental effects on immunotherapy outcomes, more research in this area may guide patient management.

**Abstract:**

Antibiotic administration is associated with worse clinical outcomes and changes to the gut microbiome in cancer patients receiving immune checkpoint inhibitors (ICI). However, the effects of antibiotics on systemic immune function are unknown. We, therefore, evaluated antibiotic exposure, therapeutic responses, and multiplex panels of 40 serum cytokines and 124 antibodies at baseline and six weeks after ICI initiation, with *p* < 0.05 and false discovery rate (FDR) < 0.2 considered significant. A total of 251 patients were included, of whom the 135 (54%) who received antibiotics had lower response rates and shorter survival. Patients who received antibiotics prior to ICI initiation had modestly but significantly lower baseline levels of nucleolin, MDA5, c-reactive protein, and liver cytosol antigen type 1 (LC1) antibodies, as well as higher levels of heparin sulfate and Matrigel antibodies. After ICI initiation, antibiotic-treated patients had significantly lower levels of MDA5, CENP.B, and nucleolin antibodies. Although there were no clear differences in cytokines in the overall cohort, in the lung cancer subset (53% of the study population), we observed differences in IFN-γ, IL-8, and macrophage inflammatory proteins. In ICI-treated patients, antibiotic exposure is associated with changes in certain antibodies and cytokines. Understanding the relationship between these factors may improve the clinical management of patients receiving ICI.

## 1. Introduction

Immune checkpoint inhibitors (ICI) have transformed the treatment landscape of many advanced cancers and have substantially improved outcomes for a subset of patients. Despite the widespread use of ICI, the identification of patients most likely to benefit from these costly and potentially toxic therapies remains challenging. Established tumor-based predictive biomarkers for ICI efficacy include PD-L1 expression, microsatellite instability, and tumor mutational burden [1,2,3,4,5]. Because ICI exerts anti-cancer effects by engaging the host immune system, systemic factors, such as patient HLA type, serum cytokines and antibodies, and circulating immune cell populations are also associated with clinical outcomes [6,7,8,9,10,11,12].

Additionally, a number of clinical characteristics appear to predict ICI efficacy. In patients with lung cancer, the extent of prior or current smoking is associated with tumor mutation burden and ICI efficacy [4]. Exposure to steroids is associated with worse outcomes, which may reflect both the immunosuppressive effects of these medications as well as a negative prognostic effect when steroids are used to palliate cancer-associated symptoms [13,14]. Overweight and obese patients appear to derive greater benefit from ICI [15], an observation that has been attributed to leptin-mediated PD-1 dysfunction, increased glutamine and other nutrients essential to immune cell development and function, production of inflammatory cytokines, and ICI dosing strategy [16,17,18,19].

Among the most striking clinical associations with ICI outcomes are antibiotic exposure. Numerous cohort studies have demonstrated that patients receiving antibiotics around the time of ICI initiation experience inferior progression-free survival (PFS) and overall survival (OS) [20,21,22,23,24,25,26]. These findings appear specific to ICI therapy and are not observed in populations receiving chemotherapy [27]. The detrimental effect of antibiotics is primarily thought to arise from changes to the gut microbiome, which plays a key role in immune function regulation and has been found to influence ICI response [28,29]. Indeed, recent studies have shown that fecal microbiota transplants may restore ICI efficacy in cases refractory to cancer immunotherapy [30,31].

Although numerous studies have established an association between antibiotics and ICI clinical outcomes, little is known about the effects of antibiotics on systemic immune parameters. We, therefore, analyzed cytokine and antibody profiles near the time of ICI initiation according to antibiotic exposure, recognizing that such observations imply association and not necessarily causation. In order to determine clinical benefit in this period, we included tumor radiographic response, which may be less prone to confounders (such as infection) than other endpoints.

## 2. Materials and Methods

### 2.1. Clinical Data Collection

This study was approved by the UT Southwestern Institutional Review Board (IRB #STU 082015-053). In this prospective cohort study, patients planned to initiate ICI for cancer therapy underwent clinical data and serial biospecimen collection. Clinical data included age, sex, race, ethnicity, cancer type and stage, ICI type, date of ICI initiation, progression-free survival (PFS), overall survival (OS), and best radiographic response (using response evaluation criteria in solid tumors [RECIST] v 1.1). We abstracted Eastern Cooperative Oncology Group (ECOG) performance status from the medical records by searching for “performance status” and “ECOG” keywords and documenting patient performance status within one month prior to or after starting ICI treatment. If performance status was not documented in this timeframe, it was designated as missing. For the current analysis, we performed a retrospective medical records review to identify antibiotic exposure. Based on earlier studies in this area, we defined antibiotic exposure as an antibiotic prescription between six weeks before and six weeks after ICI initiation [32,33,34]. We collected antibiotic name(s), date(s) of initiation, and clinical indication.

### 2.2. Biospecimen Collection and Analysis

After patients were enrolled in the cohort study, peripheral blood was collected before and 6 weeks after ICI initiation; these time-points were selected based on prior studies of baseline and post-treatment circulating immune parameters [35]. At each time-point, we collected approximately 25 mL of blood and transferred to the following tubes: ACD (Sol B) (6 mL), ACD (Sol A) (2.5 mL), and PAXgene RNA (2.5 mL). ACD tube samples were centrifuged at 1200× *g* at 4 °C for 15 min to obtain plasma.

Cytokine levels were measured using a Bio-Plex Pro Human Chemokine 40-plex Panel (Bio-Rad Laboratories, Hercules, CA, USA) on the Luminex 200 System (Supplementary Table S1). The concentration of each cytokine (pg/mL) was determined by a fit-of-curve for mean fluorescence intensity vs. pg/mL. Cytokine assays were performed in 13 batches, each of which contained approximately 80 samples. Individual patient samples and time points were clustered in the same batch. Any cytokine concentration that was flagged as significantly greater or less than the effective detection range was replaced with the accepted maximum or minimum for that batch. Cytokines with more than 10% flagged values across all batches were removed. We used the ComBat parametric empirical Bayes framework for batch correction [36]. For analysis, cytokine concentrations were displayed on a log2 scale. In the rare event that a patient had duplicate samples in a batch, we averaged the log2 value for that patient.

We previously developed and manufactured a custom protein array panel of 124 antigens, including nuclear antigens, cytosolic/matrix antigens, and tissue/organ-specific antigens (Supplementary Table S2), and have applied it to the detection of dynamic humoral immune changes after ICI initiation [37,38,39,40]. This high-throughput fluorescence-based detection system is capable of simultaneously assaying antibody reactivity to all 124 antigens with 5 μL of sera [39]. Antibody data analysis included the following pre-processing steps: (1) background subtraction and averaging of duplicated spots; (2) normalization of the signal intensity of each antigen (Ag) using internal controls across all samples; and (3) normalized signal intensity (nSI) for each Ag (Ab) completed for each Genepix Report file generated per sample [37]. Normalized fluorescence intensity (NFI) files were processed for downstream analysis using the Cluster and Treeview algorithm adopted from the Eisen Laboratory.

Antibody panels were run in six batches containing between 42 and 345 unique samples. For each antigen and batch combination, we required that at least 10% of samples had a signal-to-noise ratio ≥3. Antigens with less than 99% of values available across all samples were then dropped from analysis. Antibody data was further normalized using variance stabilizing normalization (VSN) and batch-corrected using ComBat [41]. Any values from samples that were duplicated across all batches were then averaged.

### 2.3. Statistical Analysis

For demographic characteristics, we computed *p* values using Fisher’s exact test for categorical variables and t-tests for continuous variables. We generated heat maps for baseline and 6-week cytokine and antibody values based on the z-score of each sample relative to all other samples for the given cytokine or antibody. False discovery rates (FDR) were evaluated using the Benjamini–Hochberg procedure. We produced Kaplan–Meier curves and cox regressions using R survival package (v3.1-8). We defined overall survival (OS) as the interval between ICI initiation and date of death. We defined progression-free survival (PFS) as the interval between ICI initiation and either (a) clinical progression, (b) radiographic progression, or (c) death. In the event a particular case had an unidentified endpoint, the endpoint was censored at the date of last known follow-up. For antibiotic timing comparisons, we compared individuals that received antibiotics pre-ICI to the rest of the cohort, regardless of post-ICI antibiotic exposure, and we compared individuals that received post-ICI antibiotics to the rest of the cohort, regardless of pre-ICI antibiotic exposure. Antibiotics received after ICI initiation and the cumulative number of distinct antibiotics received were modeled as time-dependent variables in Cox regressions to avoid immortality bias. Because cytokine patterns and effects differ by cancer type [42], we performed tumor-specific subset analysis. *p* values were determined using the Pearson product-moment correlation coefficient. All computation was performed with R (v3.6.3). ComBat batch correction was applied using the sva package (v3.34.0), and VSN was applied using the vsn package (v3.54.0).

## 3. Results

### 3.1. Study Cohort

A total of 251 patients were included in the analysis. Median age was 68 years and 98 (39%) were female. The most common cancer types were non-small cell lung cancer (NSCLC) (*n* = 133, 53%) and melanoma (*n* = 47, 19%). Cancer stage distribution was as follows: Stage I/II (*n* = 13; 5%); Stage III (*n* = 50; 20%); Stage IV (*n* = 161; 64%); Unknown (*n* = 27; 11%). ECOG performance status distribution was as follows: ECOG 0 (*n* = 61; 24%), ECOG 1 (*n* = 124; 49%), ECOG 2–4 (*n* = 28; 11%), and ECOG missing (*n* = 38; 15%). Overall, 135 patients (54%) received an antibiotic prescription between six weeks before and six weeks after ICI initiation as follows: 44 (18%) only prior to ICI, 57 (23%) only after ICI, and 34 (14%) both before and after ICI.

Indications for antibiotic prescriptions included the following: respiratory (*n* = 481), prophylaxis (*n* = 116), genitourinary (*n* = 94), unknown (*n* = 86), skin and soft tissue (*n* = 74), gastrointestinal (*n* = 35), sepsis (*n* = 27), other (*n* = 7), and fever (*n* = 6).

Clinical characteristics according to antibiotic exposure are shown in Table 1. There were no significant differences between patients prescribed antibiotics in the 6-week window before vs. after ICI initiation, although we observed a near-significant trend for cancer type (*p* = 0.09), with NSCLC accounting for more than 60% of cases receiving antibiotics post-ICI initiation (Supplementary Table S3).

### 3.2. Clinical Outcomes

**Figure 1:**
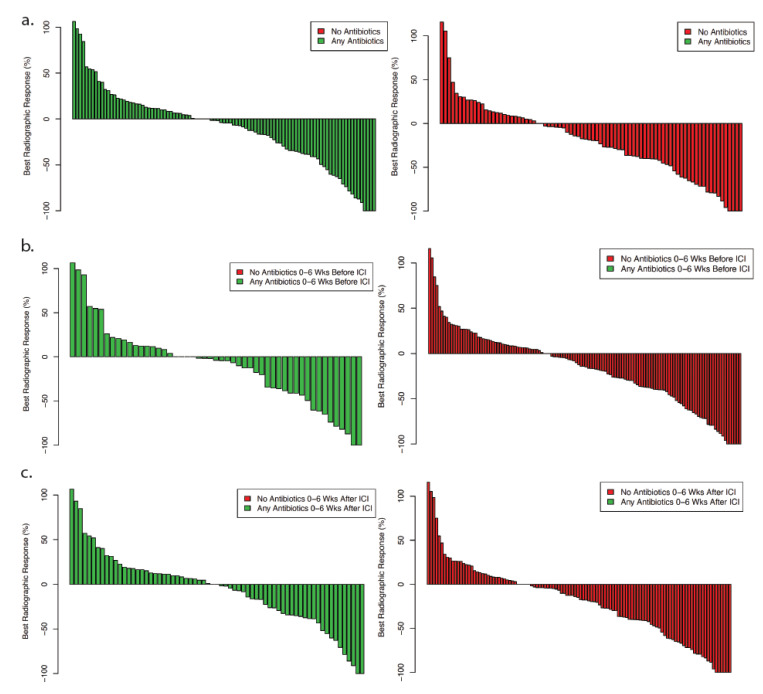
Best radiographic response according to antibiotic exposure: (**a**) any antibiotic exposure versus no antibiotic exposure; (**b**) antibiotic exposure pre-ICI initiation; (**c**) antibiotic exposure post-ICI initiation.

To establish the association between antibiotic exposure and clinical outcomes in the study cohort, we examined OS and PFS. Because these parameters may be determined well after the period of antibiotic exposure and may be subject to confounding factors, we also determined radiographic response according to response evaluation criteria in solid tumors (RECIST) (Figure 1). Evaluating radiographic response according to antibiotic exposure permitted a focus on the initial weeks after treatment initiation, representing the time period for both antibiotic exposure and biospecimen collection in this cohort. Similar to other endpoints, we observed a near-significant trend toward worse radiographic response among individuals who received antibiotics (*p* = 0.08). This difference is driven primarily by cases receiving antibiotics after ICI initiation (*p* = 0.02).

When we considered radiographic response as a categorical variable (progressive disease, PD; stable disease, SD; partial response, PR; complete response, CR), there was no association with antibiotic exposure (*p* = 0.63) (Supplementary Table S4). Although patients who received antibiotics were numerically more likely to have PD (13% versus 10%) and less likely to have PR as the best response (19% versus 24%), it is possible that small patient numbers limited statistical power.

Consistent with other reports, patients who received antibiotics had worse OS and PFS (Supplementary Figure S1 and Table S5). Similar to response rate, this effect is driven primarily by antibiotic exposure after ICI initiation.

### 3.3. Systemic Immune Parameters

After removing samples that did not meet specified quality thresholds, the availability of antibody data was as follows: baseline (*n* = 180), six weeks (*n* = 108), both time-points (*n* = 107). Availability of cytokine data was as follows: baseline (*n* = 229), six weeks (*n* = 133), both time-points (*n* = 128). After applying our statistical quality control procedure to analytes across samples, a total of 31 cytokines and 71 antibodies were suitable for further analysis.

**Figure 2:**
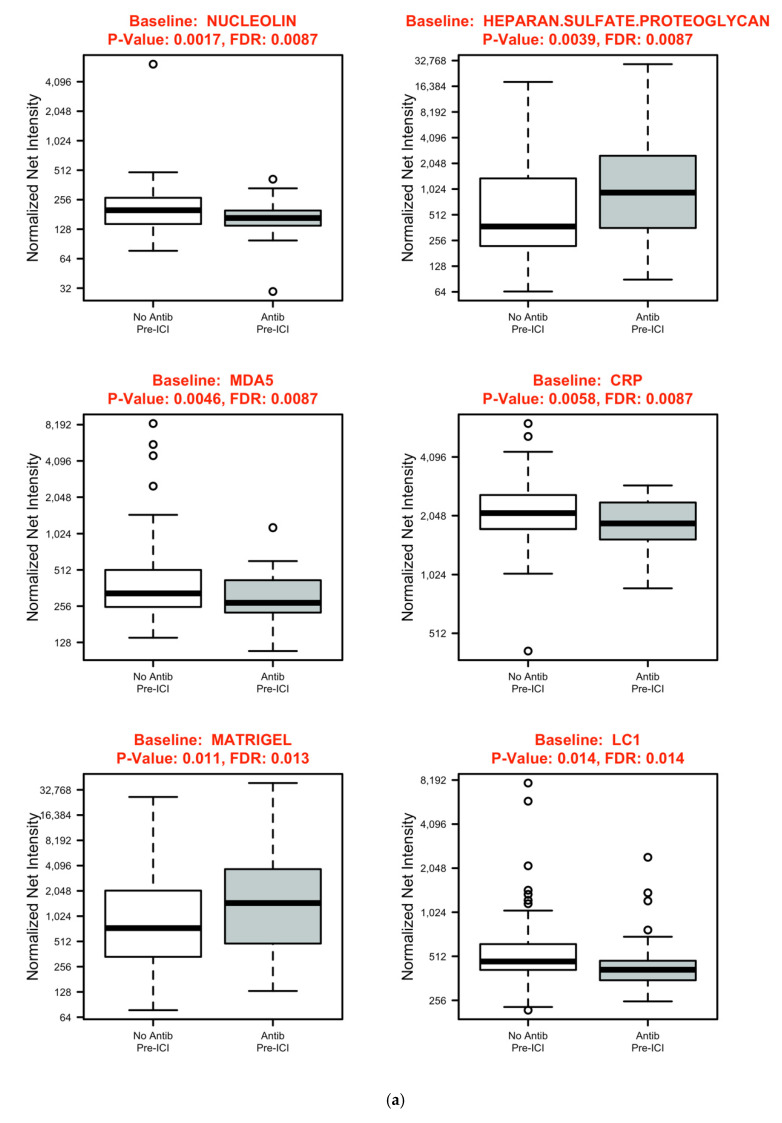

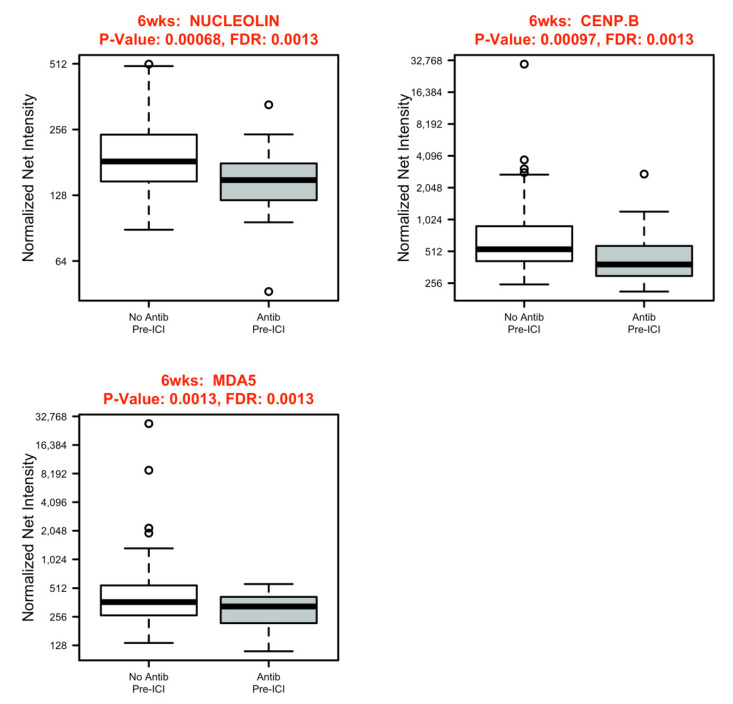
Autoantibodies with statistically significant differences (*p* < 0.05, FDR < 0.2) according to antibiotic exposure: (**a**) baseline; (**b**) 6 weeks.

**Figure 3:**
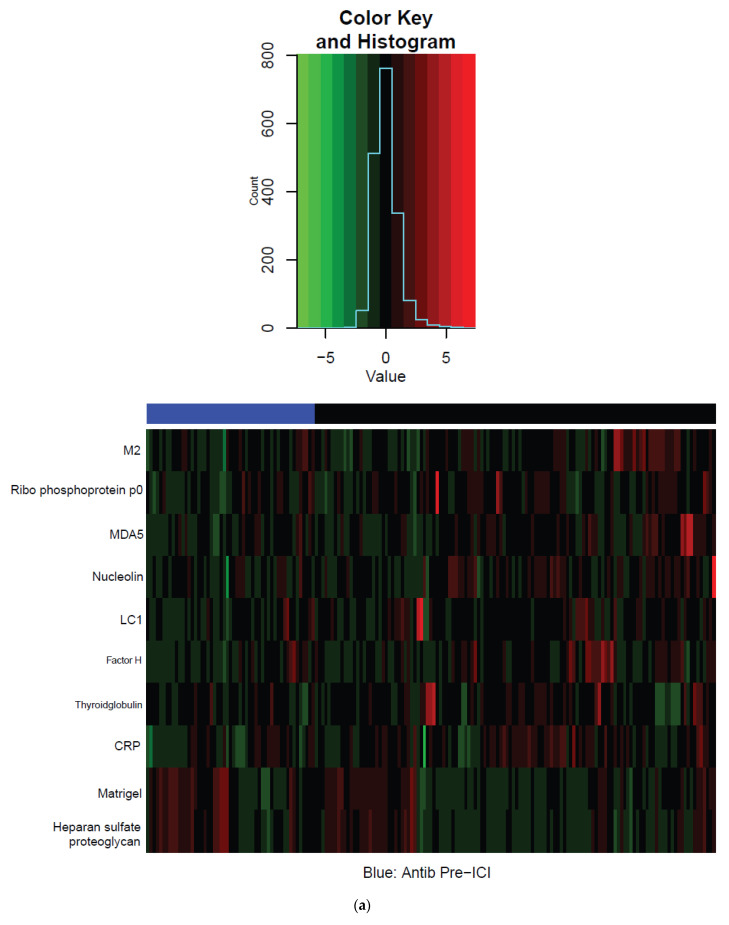

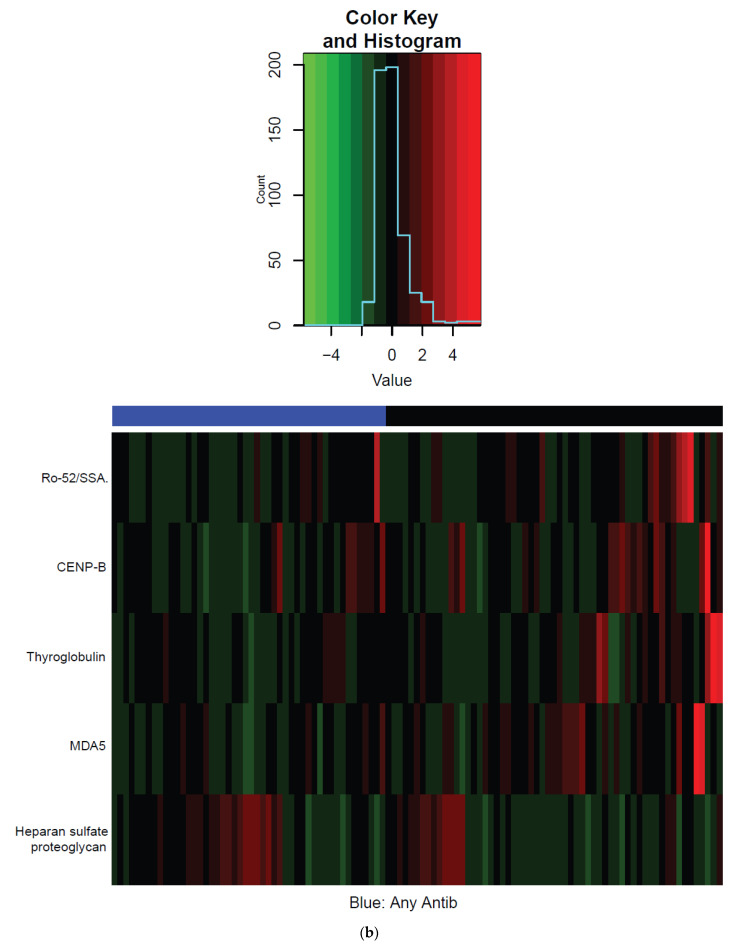
Heatmaps of antibodies with statistically significant differences (*p* < 0.05) according to antibiotic exposure: (**a**) Z–Score Baseline antibodies; (**b**) Z–Score 6 week antibodies.

As displayed in Figure 2, baseline levels of the following antibodies were modest but significantly lower in patients who received antibiotics before ICI initiation compared to other patients (all *p* < 0.05): nucleolin (FDR 0.11), C-reactive protein (CRP) (FDR 0.11), liver cytosol antigen type 1 (LC1) (FDR 0.17), and melanoma differentiation-associated protein 5 (MDA5) (FDR 0.17). Baseline levels of heparin sulfate (FDR 0.1) and Matrigel (FDR 0.16) antibodies were significantly higher in patients who received pre-ICI antibiotics. At the 6-week time-point, levels of antibodies specific for MDA5, centromere protein B (CENP-B), and nucleolin were significantly lower (all FDR 0.03) in patients who received antibiotics before starting ICI. We observed no significant differences in changes in antibody levels between the baseline and 6-week time-point. In patients receiving antibiotics after ICI initiation, there were no significant differences in antibody levels for any time permutation. Figure 3 shows summarized heatmaps of antibodies with statistically significant differences (*p* < 0.05) grouped by antibiotic exposure without incorporating the FDR cutoff. We observed no significant differences in cytokine profiles according to antibiotic exposure for any time permutation.

### 3.4. Non-Small Cell Lung Cancer (NSCLC) Subset Analysis

**Figure 4:**
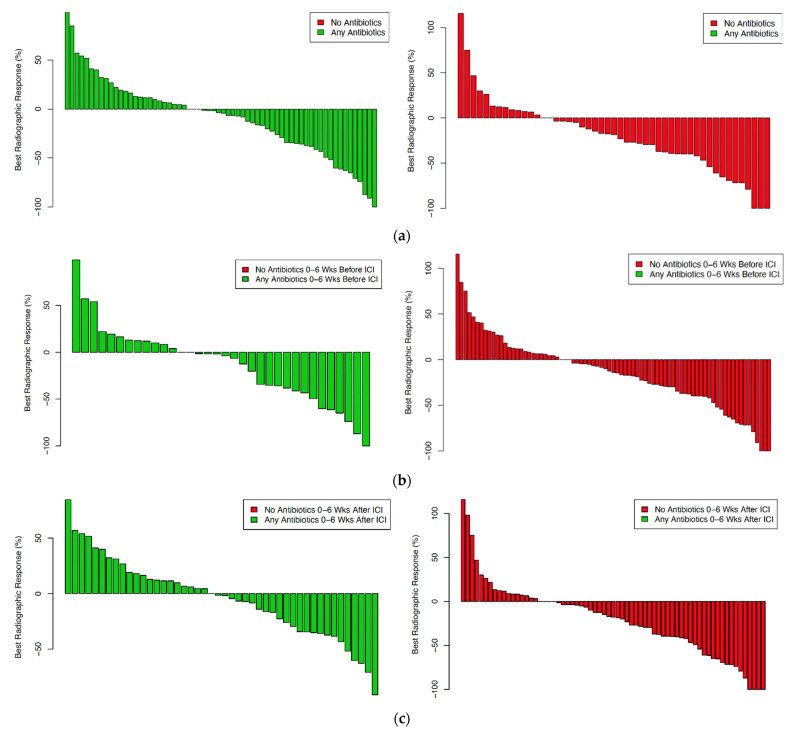
Best radiographic response according to antibiotic exposure in non-small cell lung cancer cases. (**a**) any antibiotic exposure versus no antibiotic exposure (*p* = 0.09); (**b**) antibiotic exposure pre-ICI initiation (*p* = 0.74); (**c**) antibiotic exposure post-ICI initiation (*p* = 0.02).

To eliminate the potential confounder of tumor type heterogeneity, we also analyzed the most common cancer in our cohort, the subset of NSCLC cases (N = 135). In our study cohort, NSCLC was the most prevalent type of cancer and had the highest rate of antibiotic exposure. Consistent with data from the overall cohort, we observed worse OS and a trend towards worse PFS with antibiotic exposure (Supplementary Table S5). Furthermore, radiographic response trended towards being worse with antibiotic exposure (*p* = 0.09) and was significantly worse with post-ICI antibiotic exposure (*p* = 0.02) (Figure 4). As with the overall cohort, however, we observed no association between antibiotic exposure and best RECIST response category (*p* = 0.5) (Supplementary Table S4).

**Figure 5:**
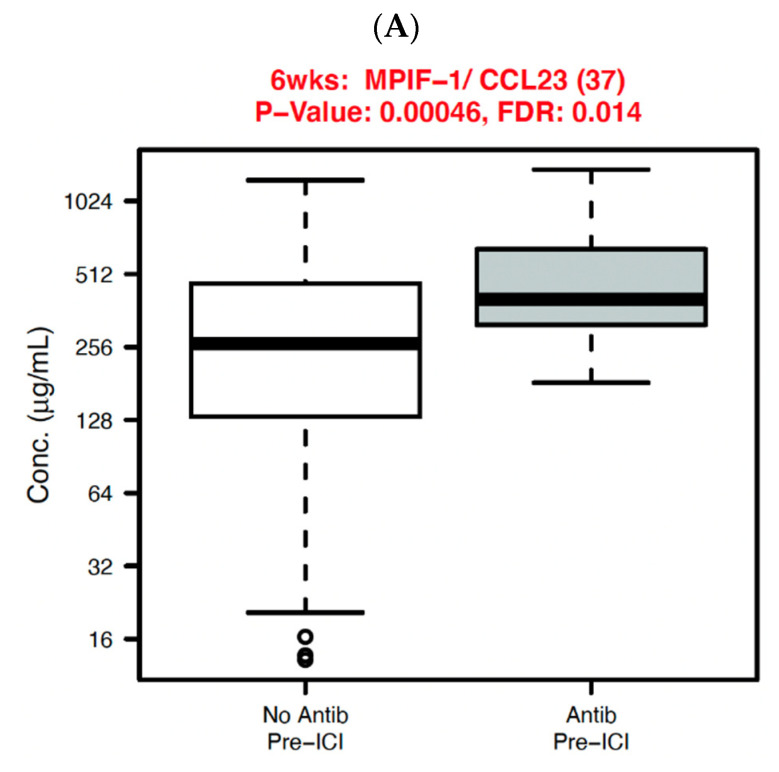

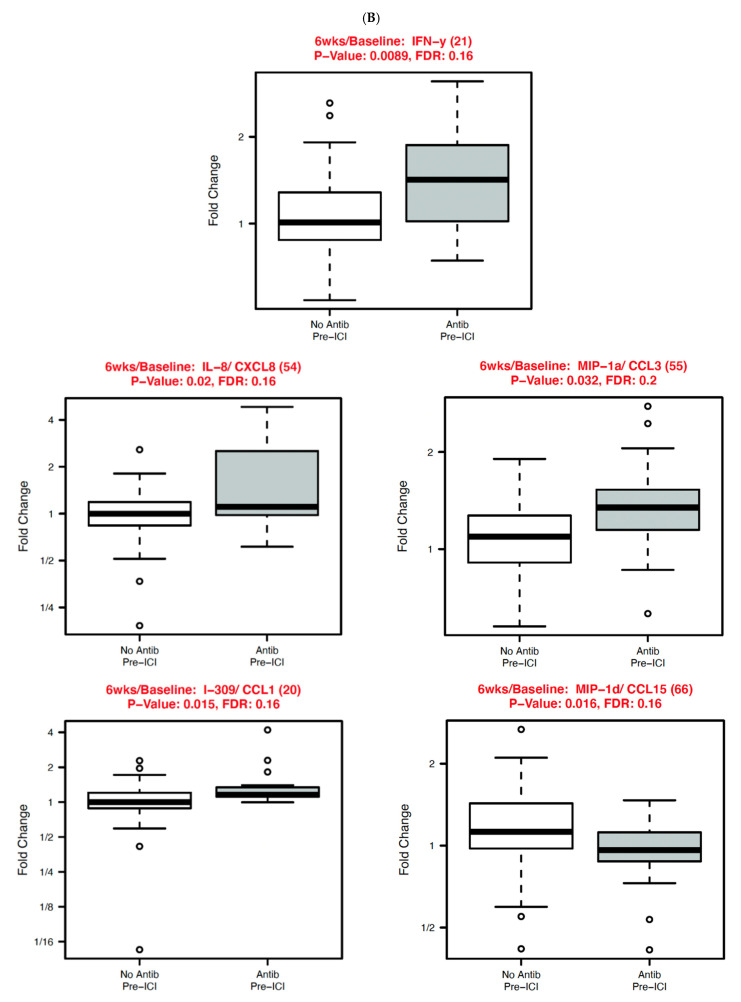
Systemic immune parameters with significant differences (*p* < 0.05 and FDR < 0.2) according to antibiotic exposure before ICI initiation in non-small cell lung cancer cases. (**A**) 6 weeks; (**B**) 6 weeks/baseline.

We analyzed systemic immune parameters in the NSCLC cohort, displaying those with significant association with antibiotic exposure in Figure 5. At six weeks, myeloid progenitor inhibitory factor-1 (MPIF-1)/chemokine (C-C motif) ligand 23 (CCL23) was significantly elevated in patients receiving pre-ICI antibiotics (*p* < 0.01, FDR 0.01). When examining the fold-change from baseline to six weeks, the following cytokines had significantly greater changes in patients with pre-ICI antibiotic exposure: interferon-gamma (IFN-γ) (*p* = 0.009, FDR 0.16), interleukin-8 (IL-8)/chemokine (C-X-C motif) ligand 8 (CXCL8) (*p* = 0.02, FDR 0.16), macrophage inflammatory protein-1a (MIP-1a)/CCL3 (*p* = 0.03, FDR 0.2), and I309/CCL1 (*p* = 0.02, FDR 0.16). The fold-change for macrophage inflammatory protein-1d (MIP-1d)/CCL15 was significantly lower in the pre-ICI antibiotic exposure group (*p* = 0.02, FDR 0.16).

We then analyzed the association between clinical outcomes and the systemic immune parameters identified as associated with antibiotic exposure. Among these, no biomarkers identified in the overall cohort were associated with outcomes, while IL-8/CXCL8 and IFN-γ were significantly associated with outcomes in the NSCLC cohort. Specifically, higher baseline IL-8/CXCL8 and fold change in IL-8/CXCL8 from baseline to six weeks was associated with inferior OS; higher fold change in IL-8/CXCL8 from baseline to six weeks was associated with inferior PFS; higher 6-week IL-8/CXCL8 was associated with inferior OS, PFS, and radiographic response; and greater positive fold-change in IFN-γ from baseline to six weeks was associated with inferior OS (Supplementary Figure S2).

## 4. Discussion

Along with smoking history, steroid exposure, and body mass index, antibiotic exposure has emerged as a clinical factor influencing ICI efficacy. Specifically, patients receiving antibiotics have consistently been found to have inferior disease control and survival, an effect attributed to changes in the gut microbiome. In the present study, we confirmed this finding and examined systemic immune parameters across ICI patient populations according to antibiotic status. We observed significant differences in levels of approximately five percent of evaluated antibodies according to antibiotic exposure.

Heparan sulfate antibody levels were higher in patients treated with antibiotics. Notably, heparan sulfate is a crucial mediator for various infections, including SARS-CoV-2 [43], suggesting that this observation may reflect the clinical condition leading to antibiotic use rather than antibiotic-induced biologic effects. We can identify no overarching function or pathway linking the other antibiotic-associated findings, namely lower levels of antibodies specific for C-reactive protein, nucleolin, MDA-5, and CENP-B. CRP is an acute-phase reactant elevated in inflammatory states and cancer [44]. Nucleolin is a nucleolar protein associated with intranucleolar chromatin. At the cell surface, it also functions as the receptor for the respiratory syncytial virus fusion protein [45]. In cancer, nucleolin may affect angiogenesis via upregulation of vascular endothelial growth factor (VEGF). MDA-5 is a receptor dsDNA helicase enzyme involved in anti-viral immunity and autoinflammation [46,47]. Anti-MDA-5 antibodies have been associated with dermatomyositis (including cancer-associated) and interstitial lung disease [48]. CENP-B is one of the three main human centromere antigenic components, and CENP-B antibodies are seen in systemic sclerosis and are upregulated in certain cancers [49].

Although we did not identify differences in cytokine profiles in the overall study cohort, in NSCLC patients—who represented almost 55% of the total study population—we noted numerous differences. We performed this tumor-specific subset analysis because cytokine patterns and effects may differ according to cancer type [42]. Among NSCLC patients, baseline levels of MPIF-1/CCL23, which inhibits T lymphocyte recruitment and function [50], were higher in the antibiotic cohort. After ICI initiation, the antibiotic-treated group also sustained greater increases in IL-8/CXCL8, a chemokine involved in myeloid leukocyte migration and neutrophil degranulation that is also associated with tumor progression and reduced benefit from immune checkpoint blockade in prior studies [9,11,51,52] and in the present analysis. We noted mixed effects on members of the MIP family, proinflammatory chemokines involved in monocyte, T lymphocyte, and dendritic cell activation [53]. We also observed greater increases in IFN-γ in the antibiotic cohort. Given the role of this cytokine in innate and adaptive immunity against viral and some bacterial infections, it is unclear whether this difference reflects antibiotic exposure or the clinical infections leading to their use.

It is well established that antibiotics alter the gastrointestinal microbiome. Antibiotic-induced dysbiosis is associated with decreased microbiome diversity, which may take years to recover [29,54]. This loss of diversity can detrimentally impact the training and maintenance of the human immune system, suggesting a potentially biologically plausible mechanism as to why antibiotic exposure correlates with reduced ICI efficacy [55]. Given this background, we hypothesized that we would detect clear differences in systemic immune parameters with antibiotic use. Why then did we observe relatively few differences in these markers? One potential explanation is that antibiotic effects on systemic immune status are manifest through different immune markers rather than antibody and cytokine profiles, such as circulating immune cell populations. Antibiotics may influence systemic antibodies and cytokines not included in our panels. Another possible explanation is that antibiotic effects on these parameters appear later than six weeks after ICI initiation. Alternatively, one might theorize that infection and antibiotics have opposing effects, thereby obscuring antibiotic-related changes. Finally, it is possible that our cytokine and antibody panels do not capture anti-tumor immune function. While we have previously demonstrated that these markers appear associated with ICI toxicity [35,37,38], their correlation with ICI efficacy is less clear.

Without clear systemic markers of antibiotic effects on anti-tumor immunity, the use of antibiotics in cancer patients undergoing immunotherapy presents a clinical conundrum. Although antibiotics have been linked to numerous adverse events, including allergic reactions, gastrointestinal toxicity, drug–drug interactions, development of antimicrobial resistance, tendinopathies, and rupture, and bleeding due to effects on colonic bacteria vitamin K production [56,57,58], antibiotic overuse remains prevalent. Indeed, it is estimated that at least 30% of patients with the common cold are provided with antibiotic prescriptions in the U.S., an unnecessary practice [59]. There is no clear approach to studying this question prospectively. Withholding antibiotics in patients with infections or administering antibiotics to patients with no clinical indication—particularly since it appears to worsen ICI efficacy—is not ethical. For now, clinicians will need to approach these cases individually. Given the high rates of antibiotic use in oncology populations, they are likely to encounter these scenarios relatively frequently [25].

In the present study, antibiotic exposure prior to ICI initiation had the strongest association with systemic immune parameters, while antibiotic exposure after ICI initiation had the strongest association with clinical outcomes. While the reasons for this discrepancy cannot be determined from available data, there are some potential explanations. First, the cohort receiving antibiotics prior to ICI initiation was smaller than the antibiotics post-ICI initiation cohort. While this cohort demonstrated a trend toward worse clinical outcomes, a small sample size may have limited statistical power. Another potential factor is the nature of the cohort receiving antibiotics post-ICI initiation. Because of the growing awareness of antibiotics’ detrimental effects on clinical outcomes in patients receiving checkpoint inhibitor therapy, it is possible that clinicians may now be hesitant to prescribe the agents, resulting in their use only in more clinically severe cases, which in turn might have worse outcomes. Lastly, the effects of post-ICI antibiotics on systemic immune parameters may not be apparent during the assessment period. The median time to maximal effect of antibiotics on the gut microbiome is nine days [60]. If microbiome changes mediate the association between antibiotic exposure and changes in systemic immune parameters, the process may take even longer. As a result, these changes may not be apparent by the 6-week time-point in patients who receive antibiotics post-ICI initiation.

This study has a number of limitations. First, this is a retrospective study from a single institution. Second, data for additional factors that may alter the gastrointestinal microbiome, such as diet, country of origin, medications other than antibiotics, and genetic predisposition, were not available. We also recognize that our review of clinical data may have missed antibiotic prescriptions. Conversely, patients may have been prescribed but never taken antibiotics. While we were able to identify the clinical indication for most antibiotic prescriptions, it is not possible to determine the association between indication, systemic immune parameters, or clinical outcomes because patients may have received multiple antibiotics. Importantly, we do not have stool samples to assess the gut microbiome in study subjects. Finally, in this study, we are unable to determine whether the observed associations between antibiotic exposure and immune parameters represent causative effects or surrogates for other phenomena such as clinical infections or tumor-associated inflammatory changes. Such distinction might require a prospective, randomized trial of antibiotics in patients receiving ICI, which is unlikely to be feasible.

Despite these limitations, we note certain strengths, including the availability of pre- and post-treatment plasma samples, a cohort large enough to confirm the established effect of antibiotics on ICI outcomes, and extensively characterized clinical data.

## 5. Conclusions

To our knowledge, our assessment of cytokines and antibodies represents the first interrogation of systemic immune parameters according to antibiotic exposure in a population treated with ICI. Although antibiotics were associated with inferior clinical outcomes, including radiographic response, they were associated with few differences in cytokine and antibody profiles. Furthermore, these differences did not reveal any patterns with clear clinical or biological significance. Mechanistic studies, including stool composition and immune cell population analysis, might further elucidate the molecular effects of antibiotic exposure on immune physiology. Increased understanding of the relationship between these factors may allow clinicians to improve the clinical management of patients receiving ICI.

## 6. Patents

Drs. Khan, Fattah, Park, Xie, Li, Wakeland, and Gerber report a U.S. patent application (62/654,025).

## Figures and Tables

**Table 1:** Clinical characteristics according to antibiotic exposure.

Characteristic	Total	Antibiotics	No Antibiotics	*p* Value
	Median (Range) or N (%)	Median (Range) or N (%)	Median (Range) or N (%)	
**Age (years)**	68 (29–2)	67 (29–86)	68 (35–92)	0.27
**Gender**				0.8
Female	98 (39)	54 (40)	44 (38)	
Male	153 (61)	81 (60)	72 (62)	
**Race-ethnicity**				0.23
Non-Hispanic white	193 (77)	108 (80)	85 (73)	
Other	58 (23)	27 (20)	31 (27)	
**Cancer type**				0.26
NSCLC	133 (53)	73 (54)	60 (52)	
Melanoma	47 (19)	29 (21)	18 (16)	
Other	71 (28)	33 (24)	38 (33)	
**BMI**				0.61
<25	103 (41)	53 (39)	50 (43)	
≥25	147 (59)	81 (60)	66 (57)	
**Receipt of anti-CTLA4**				0.33
No	221 (88)	116 (86)	105 (91)	
Yes	30 (12)	19 (14)	11 (9)	
**ECOG Performance status**				0.11
0–1	185 (74)	95 (70)	90 (78)	
2–4	28 (11)	19 (14)	9 (8)	
Missing	38 (15)	21 (16)	17 (14)	
**Cancer Stage**				0.29
I/II	13 (5)	6 (4)	7 (6)	
III	50 (20)	32 (24)	18 (16)	
IV	161 (64)	84 (62)	77 (66)	
Missing	27 (11)	13 (10)	14 (12)	

BMI, body mass index; CTLA4, cytotoxic T lymphocyte antigen 4; ECOG, Eastern Cooperative Oncology Group; NSCLC, non-small cell lung cancer.

## Data Availability

The data presented in this study are available on reasonable request from the corresponding author. The data are not publicly available due to presence of Protected Health Information (PHI).

## Supplementary Materials

The following supporting information can be downloaded at: https://www.mdpi.com/article/10.3390/cancers14051327/s1, Figure S1: Clinical outcomes ac-cording to antibiotic exposure, Table S1: List of cytokines in multi-plex panel, Table S2: List of antibodies in multiplex panel. Table S3: Clinical characteristics according to timing of antibiotic exposure, Table S4: Association between antibiotic exposure and best RECIST category, Table S5: Progression-free and overall survival Cox regression, Table S6: Progression-free and overall survival Cox regression in non-small cell lung cancer cases.
