# Supplementary material for: Association between Antibiotic Exposure and Systemic Immune Parameters in Cancer Patients Receiving Checkpoint Inhibitor Therapy
Source: Cancers (Basel). 2022 Mar 4;14(5):1327. doi: 10.3390/cancers14051327 (PMC8909108; doi:10.3390/cancers14051327)
Supplement: Supplementary file 1 [file cancers-14-01327-s001.zip › cancers-1577581-supplementary.pdf]

Supplemental material for "Association between antibiotic exposure and systemic immune parameters in cancer patients receiving checkpoint inhibitor therapy."

Mitchell S. von Itzstein[1,2]†, Amrit S. Gonugunta[3]†, Thomas Sheffield[4], Jade Homsi[1,2], Jonathan E. Dowell[1,2], Andrew Y. Koh[5], Prithvi Raj[6], Farjana Fattah[2], Yiqing Wang[4], Vijay S. Basava[2], Shaheen Khan[6], Jason Y. Park[7], Vinita Popat[3], Jessica M. Saltarski[2], Yvonne Gloria-McCutchen[2], David Hsiehchen[1,2], Jared Ostmeyer[4], Yang Xie[2,4], Quan-Zhen Li[6], Edward K. Wakeland[6] and David E. Gerber[1,2,4]*

[1]Department of Internal Medicine (Division of Hematology-Oncology)
[2]Harold C. Simmons Comprehensive Cancer Center
[3]School of Medicine
[4]Department of Population and Data Sciences
[5]Department of Pediatrics
[6]Department of Immunology
[7]Department of Pathology; University of Texas Southwestern Medical Center. Dallas, Texas. USA
†These authors contributed equally
*Correspondence: david.gerber@utsouthwestern.edu

Contents

Supplemental Table S1

Supplemental Table S2

Supplemental Table S3

Supplemental Table S4

Supplemental Table S5

Supplemental Table S6

Supplemental Figure S1

Supplemental Figure S2

Supplemental Tables

Supplemental Table S1. List of cytokines in multiplex panel.

Total	Passed Quality Control
6Ckine/ CCL21	6Ckine/ CCL21
BCA-1/ CXCL13	BCA-1/ CXCL13
CTACK/ CCL27	CTACK/ CCL27
ENA-78/ CXCL5	Eotaxin/ CCL11
Eotaxin/ CCL11	Eotaxin-2/ CCL24
Eotaxin-2/ CCL24	Fractalkine/ CX3CL1
Eotaxin-3/ CCL26	Gro-a/ CXCL1
Fractalkine/ CX3CL1	Gro-b/ CXCL2
GCP-2/ CXCL6	I-309/ CCL1
GM-CSF	IFN-y
Gro-a/ CXCL1	IL-4
Gro-b/ CXCL2	IL-8/ CXCL8
I-309/ CCL1	IL-10
IFN-y	IL-16
IL-1b	IP-10/ CXCL10
IL-2	I-TAC/ CXCL11
IL-4	MCP-1/ CCL2
IL-6	MCP-2/ CCL8
IL-8/ CXCL8	MCP-4/ CCL13
IL-10	MDC/ CCL22
IL-16	MIF
IP-10/ CXCL10	MIG/ CXCL9
I-TAC/ CXCL11	MIP-1a/ CCL3
MCP-1/ CCL2	MIP-1d/ CCL15
MCP-2/ CCL8	MIP-3a/ CCL20
MCP-3/ CCL7	MIP-3b/ CCL19
MCP-4/ CCL13	MPIF-1/ CCL23

MDC/ CCL22	SCYB16/ CXCL16
MIF	SDF-1a+b/ CXCL12
MIG/ CXCL9	TECK/ CCL25
MIP-1a/ CCL3	TNF-a
MIP-1d/ CCL15	
MIP-3a/ CCL20	
MIP-3b/ CCL19	
MPIF-1/ CCL23	
SCYB16/ CXCL16	
SDF-1a+b/ CXCL12	
TARC/ CCL17	
TECK/ CCL25	
TNF-a	

Supplemental Table S2. List of antibodies in multiplex panel.

Total			
S100	Cytochrome C	Histone H4	Prothrombin protein
Aggrecan	Decorin-bovine	Insulin	Ribo Phosphoprotein P0
AGTR	DGPS	Intrinsic Factor	Ribo Phosphoprotein P1
alpha-actinine	DNA Polymerase beta (POLB)	Jo-1	Ribo Phosphoprotein P2
Alpha Fodrin	dsDNA	Ku (P70/P80)	Ro/SSA (52 Kda)
Amyloid	EBNA1	La/SSB	Ro/SSA (60 Kda)
AQP4	Elastin	Laminin	Scl-70/Topoisomerase I
B2-microglobulin	Entaktin EDTA	LC1	Sm
B2 glycoprotein 1	Factor I	LKM1	Sm/RNP
BAFF	Factor P	LPS	SmD
BPI	FactorB	M2	SmD1,D2,D3
Cardolipin	FactorD	Matrigel	SP100
CENP-A	FactorH	MDA5	Sphingomyelin
CENP-B	Fibrinogen IV	Mi-2	SRP54
Chondroitin Sulfate C	Fibrinogen S	Mitochondrial antigen	ssDNA
Chromatin	Fibronectin	MPO	ssRNA
Collagen I	GAD65	Muscarinic receptor	T1F1 gama
Collagen II	GBM	Myelin-associated glycoprotein	Thyroglobulin
Collagen III	Genomic DNA	Myelin basic protein (MBP)	TNFa
Collagen IV	Gliadin	Myosin	Topoisomerase I
Collagen V	Glycated Albumin-human	Nucleolin	TPO
Collagen VI	Glycyl-tRNA Synthetase (EJ)	Nucleosome	TTG
complement C1q	GP210	Nup 62	U1-snRNP 68/70
complement C3/C3a	GP2	PCNA	U1-snRNP A
complement C3b	Hemocyanin	Peroxiredoxin 1	

complement C4<br>complement C5<br>complement C6<br>complement C7<br>complement C8<br>complement C9<br>CRP	Heparan sulfate proteoglycan<br>Heparan Sulphate<br>Heparin<br>Core Histone<br>Histone H1<br>Histone H2A<br>Histone H2B<br>Histone H3	Phophatidylinositol<br>PL-7<br>PL-12<br>PM/Scl-75<br>PM/Scl 100<br>POLB<br>PR3<br>Proteoglycan	U1-snRNP B/B'<br>U1-snRNP C<br>Vimentin<br>Vitronectin
**Passed Quality Control**			
AGGRECAN<br>AGTR<br>ALPHA.FODRIN<br>AQP4<br>BPI<br>CENP.B<br>COLLAGEN.IV<br>COMPLEMENT.C1Q<br>COMPLEMENT.C3.C3A<br>COMPLEMENT.C4<br>COMPLEMENT.C5<br>COMPLEMENT.C6<br>COMPLEMENT.C7<br>COMPLEMENT.C8	EBNA1<br>ELASTIN<br>ENTAKTIN.EDTA<br>FACTOR.I<br>FACTORB<br>FACTORH<br>FIBRINOGEN.IV<br>FIBRINOGEN.S<br>FIBRONECTIN<br>GLIADIN<br>GP2<br>HEPARAN.SULFATE PROTEOGLYCAN<br>HISTONE.H1<br>HISTONE.H2B<br>INTRINSIC.FACTOR<br>JO.1<br>KU..P70.P80.<br>LA.SSB	LC1<br>M2<br>MATRIGEL<br>MDA5<br>MI.2<br>MITOCHONDRIAL.ANTIGEN<br>MPO<br>MUSCARINIC.RECEPTOR<br>MYOSIN<br>NUCLEOLIN<br>NUCLEOSOME<br>PCNA<br>PEROXIREDOXIN.1<br>PL.12	PL.7<br>PM.SCL.100<br>RIBO.PHOSPHOPROTEIN.P0<br>RIBO.PHOSPHOPROTEIN.P1<br>RIBO.PHOSPHOPROTEIN.P2<br>RO.SSA..52.KDA.<br>SCL.70.TOPOISOMERASE.I<br>SM<br>SMD<br>SMD1.D2.D3<br>SRP54<br>SSDNA<br>T1F1.GAMA<br>THYROGLOBULIN<br>TPO<br>TTG<br>U1.SNRNP.68.70<br>U1.SNRNP.A

COMPLEMENT.C9<br><br>CORE.HISTONE<br><br>CRP<br><br>DSDNA			U1.SNRNP.B.B.<br><br>U1.SNRNP.C<br><br>VITRONECTIN

Supplemental Table S3. Clinical characteristics according to timing of antibiotic exposure

**Characteristic**	**Total**<br><br>**Median (range) or N (%)**	**Antibiotic exposure only 0-6 weeks prior to ICI Median (range) or N (%)**	**Antibiotic exposure only 0-6 weeks after ICI Median (range) or N (%)**	***P* value**
**Age (years)**	68 (29-92)	66.5 (29-82)	67 (33-83)	0.3
**Gender**				
Female	98 (39)	16 (36)	21 (37)	1
Male	153 (61)	28 (64)	36 (63)	
**Race-ethnicity**				
Non-Hispanic white	193 (77)	34 (77)	45 (79)	1
Other	58 (23)	10 (23)	12 (21)	
**Cancer type**				
NSCLC	133 (53)	19 (43)	35 (61)	0.09
Melanoma	47 (19)	14 (32)	8 (14)	
Other	71 (28)	11 (25)	14 (25)	
**BMI**				
< 25	103 (41)	16 (36)	25 (44)	0.42
≥ 25	147 (59)	28 (64)	31 (54)	
**Receipt of anti-CTLA4**				
No	221 (88)	40 (91)	50 (88)	0.75
Yes	30 (12)	4 (9)	7 (12)	

BMI, body mass index; CTLA4, cytotoxic T lymphocyte antigen 4; NSCLC, non-small cell lung cancer.

Supplemental Table S4. Association between antibiotic exposure and best RECIST response category.

Overall cohort

RECIST Category	Total n (%)	Any antibiotics n (%)	No antibiotics n (%)	*P*
PD	29 (12)	17 (13)	12 (10)	0.63
SD	89 (35)	51 (38)	38 (33)	
PR	53 (21)	25 (19)	28 (24)	
CR	8 (3)	4 (3)	4 (3)	

Non-small cell lung cancer cohort

RECIST Category	Total n (%)	Any antibiotics n (%)	No antibiotics n (%)	*P*
PD	16 (12)	11 (15)	5 (8)	0.5
SD	60 (45)	34 (47)	26 (43)	
PR	33 (25)	18 (25)	15 (25)	
CR	4 (3)	1 (1)	3 (5)	

CR, complete response; PD, progressive disease; PR, partial response; RECIST, Response Evaluation Criteria in Solid Tumors; SD, stable disease.

Supplemental Table S5. Progression-free and overall survival Cox regression.

Progression-free survival				
**Covariate**	**Univariate analysis**		**Multivariate analysis**	
	**HR (95% CI)**	***P* value**	**HR (95% CI)**	***P* value**
**Age (continuous variable)**	1.01 (1-1.03)	0.094	0.99 (0.97-1.01)	0.56
**Antibiotic exposure**				
No antibiotics	Reference			
Any Antibiotics	1.48 (1.06-2.06)	0.02	*	*
Antibiotics 0-6 weeks before ICI	1.29 (0.89-1.85)	0.17	1.24 (0.81-1.9)	0.33
Antibiotics 0-6 weeks after ICI	1.8 (1.28-2.53)	<0.001	1.61 (1.06-2.45)	0.026
**Number of unique antibiotics (each additional)**	1.2 (1.1-1.31)	<0.001	*	*
**BMI**				
<25	Reference			
≥ 25	0.68 (0.48-0.94)	0.02	0.77 (0.51-1.15)	0.2
**Male gender**				
Female	Reference			
Male	1.04 (0.74-1.47)	0.81	1.32 (0.87-2.03)	0.2
**Cancer type**				
Other	Reference			
NSCLC	1.03 (0.71-1.5)	0.86	1.15 (0.74-1.8)	0.53
Melanoma	0.41 (0.22-0.75)	0.004	0.42 (0.19-0.93)	0.033
**Anti-CTLA-4**				
Not received	Reference			
Received	1.23 (0.73-2.08)	0.43	1.28 (0.64-2.56)	0.48
**Cancer stage**				
Stage I-II	Reference			
Stage III	1.13 (0.46-2.77)	0.79	1.23 (0.48-3.14)	0.66
Stage IV	1.98 (0.87-4.53)	0.1	1.64 (0.7-3.83)	0.25
**ECOG performance status**				
0-1	Reference			
2-4	2.09 (1.3-3.34)	0.002	1.34 (0.76-2.35)	0.31

Overall survival				
**Covariate**	**Univariate analysis**		**Multivariate analysis**	
	**HR (95% CI)**	***P* value**	**HR (95% CI)**	***P* value**
**Age (continuous variable)**	1.02 (1-1.04)	0.01	1.01 (0.99-1.03)	0.36
**Antibiotic exposure**				
No antibiotics	Reference			
Any Antibiotics	1.7 (1.22-2.36)	0.002	*	*
Antibiotics 0-6 weeks before ICI	1.12 (0.79-1.61)	0.52	1.12 (0.74-1.72)	0.59
Antibiotics 0-6 weeks after ICI	2.32 (1.67-3.23)	<0.001	2.35 (1.57-3.53)	<0.001
**Number of unique antibiotics (each additional)**	1.27 (1.17-1.37)	<0.001	*	*
**BMI**				
<25	Reference			
≥ 25	0.7 (0.51-0.97)	0.034	0.88 (0.6-1.29)	0.5
**Gender**				
Female	Reference			
Male	1.05 (0.75-1.46)	0.8	1.39 (0.92-2.09)	0.12
**Cancer type**				
Other	Reference			
NSCLC	1.33 (0.91-1.94)	0.14	1.44 (0.91-2.27)	0.12
Melanoma	0.42 (0.23-0.8)	0.008	0.42 (0.19-0.96)	0.039
**Anti-CTLA-4**				
Not received	Reference			
Received	0.91 (0.54-1.54)	0.73	1.06 (0.55-2.04)	0.86
**Cancer stage**				
Stage I-II	Reference			
Stage III	0.88 (0.37-2.09)	0.77	0.78 (0.31-1.97)	0.61
Stage IV	1.62 (0.75-3.5)	0.21	1.27 (0.58-2.81)	0.55
**ECOG performance status**				
0-1	Reference			
2-4	2.45 (1.53-3.93)	<0.001	1.39 (0.78-2.46)	0.26

BMI, body mass index; CTLA4, cytotoxic T lymphocyte antigen 4; ECOG, Eastern Cooperative Oncology Group; HR, hazard ratio; ICI, immune checkpoint inhibitor; NSCLC, non-small cell lung cancer.

*Not calculated as highly correlated to antibiotics before and after ICI

Supplemental Table S6. Progression-free and overall survival Cox regression in non-small cell lung cancer cases.

**Progression-free survival**				
**Covariate**	**Univariate analysis**		**Multivariate analysis**	
	**HR (95% CI)**	***P* value**	**HR (95% CI)**	***P* value**
**Age (continuous variable)**	0.99 (0.97-1.04)	0.64	0.99 (0.96-1.02)	0.55
**Antibiotic exposure**				
No antibiotics	Reference			
Any Antibiotics	1.37 (0.89-2.09)	0.15	*	*
Antibiotics 0-6 weeks before ICI	1.31 (0.81-2.12)	0.27	1.15 (0.68-1.95)	0.61
Antibiotics 0-6 weeks after ICI	1.49 (0.96-2.33)	0.08	1.63 (0.96-2.77)	0.069
**Number of unique antibiotics (each additional)**	1.15 (1.03-1.28)	0.01	*	*
**BMI**				
<25	Reference			
≥ 25	0.71 (0.46-1.09)	0.12	0.72 (0.44-1.18)	0.19
**Male gender**				
Female	Reference			
Male	1.2 (0.78-1.85)	0.41	1.34 (0.82 - 2.22)	0.25
**Anti-CTLA-4**				
Not received	Reference			
Received	0.93 (0.23-3.81)	0.92	0.77 (0.18-3.29)	0.72
**Cancer stage**				
Stage I-II	Reference			
Stage III	1.06(0.39-2.89)	0.91	0.92(0.32-2.67)	0.88
Stage IV	1.32(0.53-3.3)	0.55	1.2(0.47-3.08)	0.71
**ECOG performance status**				
0-1	Reference			
2-4	1.76(0.93-3.35)	0.084	1.47(0.75-2.9)	0.27

Overall survival				
**Covariate**	**Univariate analysis**		**Multivariate analysis**	
	**HR (95% CI)**	***P* value**	**HR (95% CI)**	***P* value**
**Age (continuous variable)**	1 (0.98-1.02)	0.84	1.01 (0.98 - 1.03)	0.58
**Antibiotic exposure**				
No antibiotics	Reference			
Any Antibiotics	1.83 (1.21-2.76)	<0.01	*	*
Antibiotics 0-6 weeks before ICI	1.4 (0.88-2.22)	0.2	1.23 (0.73-2.05)	0.44
Antibiotics 0-6 weeks after ICI	1.93 (1.27-2.93)	<0.01	2.75 (1.67-4.54)	<0.001
**Number of unique antibiotics (each additional)**	1.22 (1.12-1.33)	<0.01	*	*
**BMI**				
<25	Reference			
≥ 25	0.8 (0.53-1.21)	0.29	0.84 (0.53-1.34)	0.46
**Gender**				
Female	Reference			
Male	1.23 (0.82-1.86)	0.32	1.39 (0.87-2.23)	0.17
**Anti-CTLA-4**				
Not received	Reference			
Received	0.59 (0.14-2.39)	0.46	0.53 (0.13-2.18)	0.38
**Cancer stage**				
Stage I-II	Reference			
Stage III	0.69 (0.28-1.72)	0.43	0.51 (0.19-1.36)	0.18
Stage IV	0.96(0.44-2.1)	0.93	0.82 (0.37-1.85)	0.63
**ECOG performance status**				
0-1	Reference			
2-4	2.1 (1.11-3.97)	0.023	1.79 (0.92-3.5)	0.089

BMI, body mass index; CTLA4, cytotoxic T lymphocyte antigen 4; ECOG, Eastern Cooperative Oncology Group; HR, hazard ratio; ICI, immune checkpoint inhibitor; NSCLC, non-small cell lung cancer.

*Not calculated as highly correlated to antibiotics before and after ICI

Supplemental Figure S1.

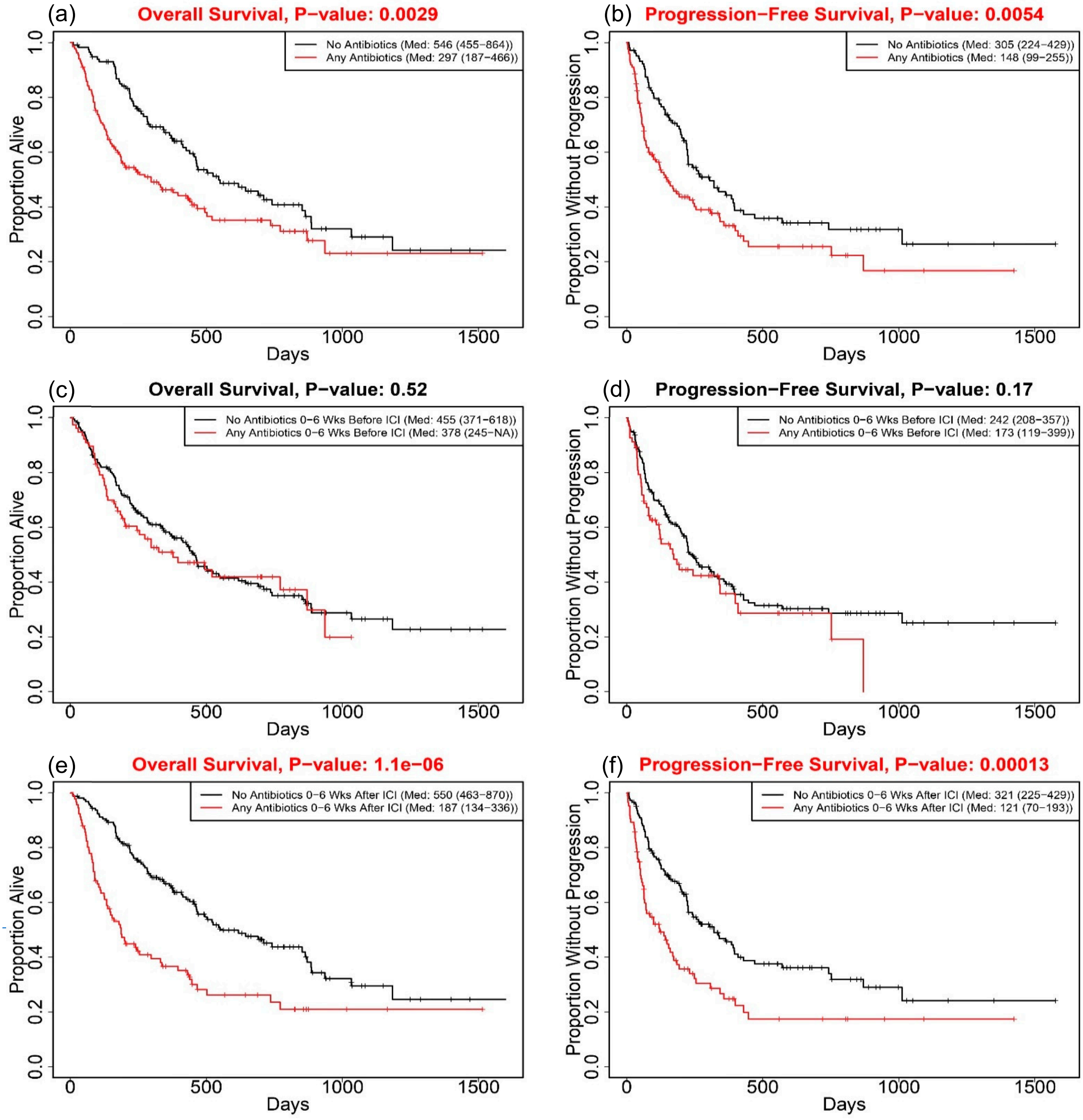

Supplemental Figure S1. Clinical outcomes according to antibiotic exposure. ***(a)*** OS, any antibiotic exposure; ***(b)*** PFS, any antibiotic exposure; ***(c)*** OS, antibiotic exposure pre-ICI initiation; ***(d)*** PFS, antibiotic exposure pre-ICI initiation; ***(e)*** OS, antibiotic exposure post-ICI initiation; ***(f)*** PFS, antibiotic exposure post-ICI initiation

Supplemental Figure S2.

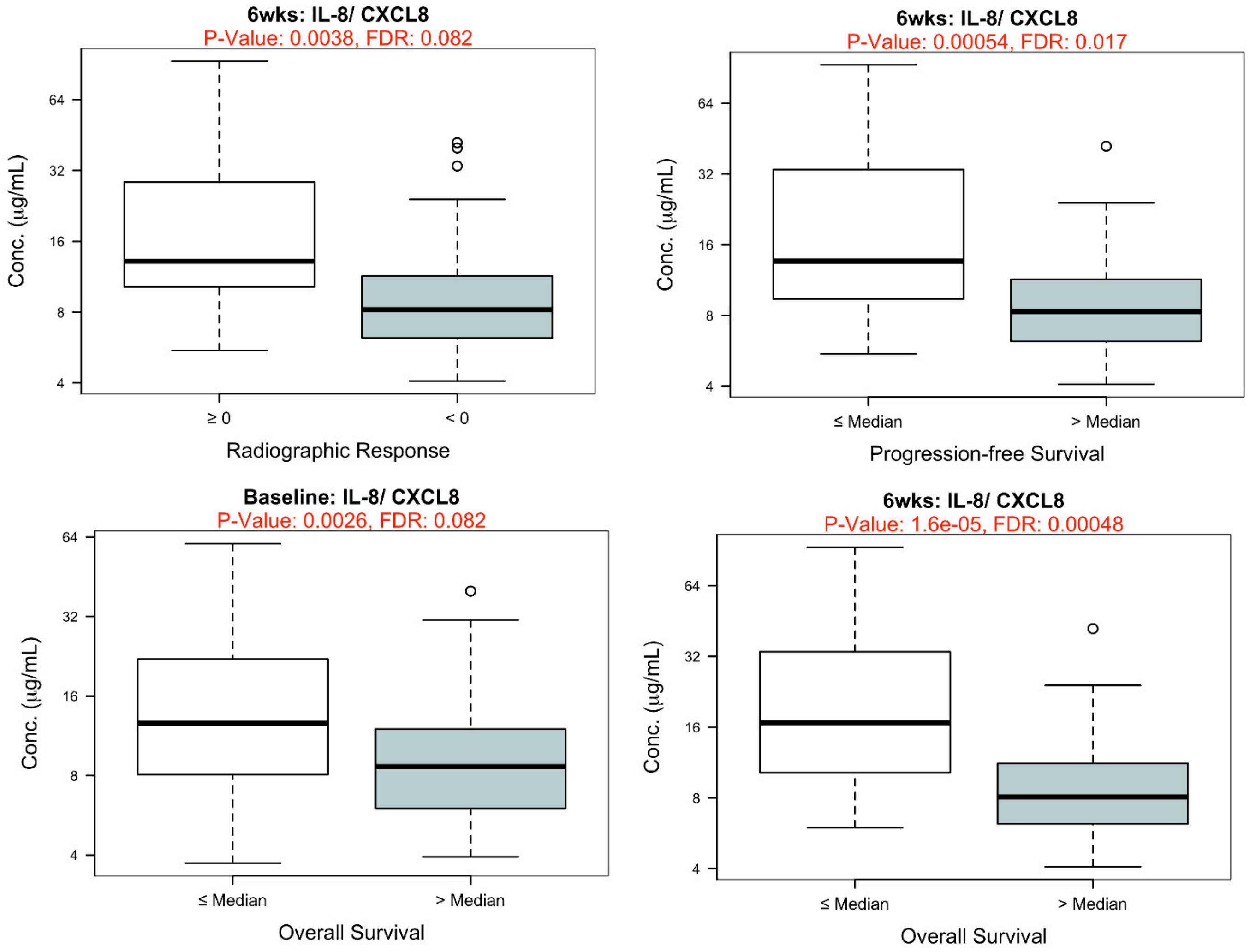

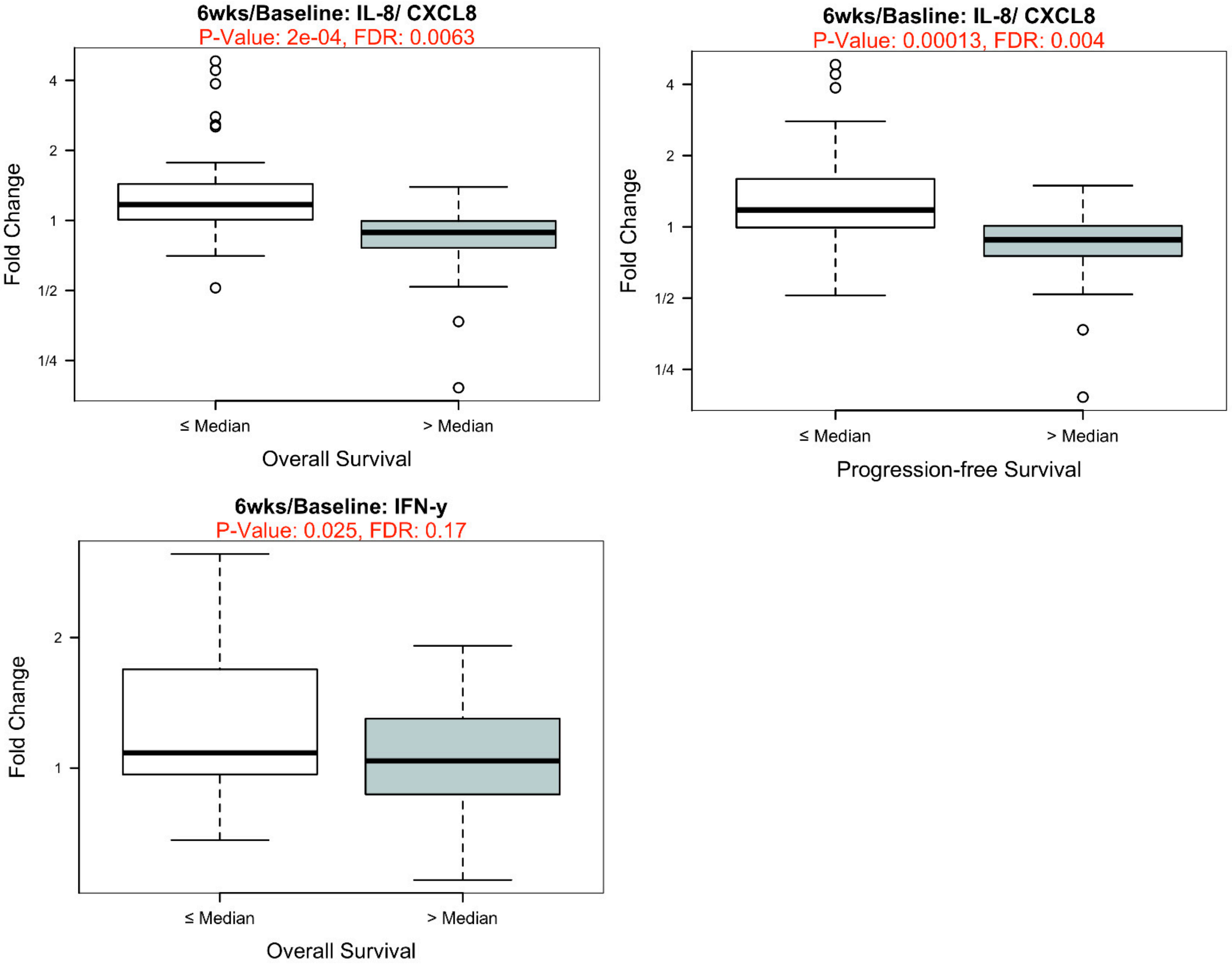

Supplemental Figure S2. Significant associations between clinical outcomes and the systemic immune parameters identified as associated with antibiotic exposure in the non-small cell lung cancer cohort.
